# The use of spatial and genetic tools to assess *Plasmodium falciparum* transmission in Lusaka, Zambia between 2011 and 2015

**DOI:** 10.1186/s12936-020-3101-7

**Published:** 2020-01-15

**Authors:** Daniel J. Bridges, Sandra Chishimba, Mulenga Mwenda, Anna M. Winters, Erik Slawsky, Brenda Mambwe, Conceptor Mulube, Kelly M. Searle, Aves Hakalima, Roy Mwenechanya, David A. Larsen

**Affiliations:** 1 PATH MACEPA, National Malaria Elimination Centre, Gt East Rd, Lusaka, Zambia; 2 Akros, 45A Roan Road, Lusaka, Zambia; 3 School of Public and Community Health Sciences, University of Montana, Missoula, MT USA; 4 Department of Public Health, Syracuse University, Syracuse, NY USA; 5 School of Public Health, University of Minnesota, Minneapolis, MN USA; 6 Lusaka District Health Management Team, Ministry of Health, Lusaka, Zambia; 7 Department of Biomedical Sciences, School of Veterinary Medicine, University of Zambia, Lusaka, Zambia

**Keywords:** Malaria, *Plasmodium falciparum*, Urban transmission, Importation, Genetic analysis

## Abstract

**Background:**

Zambia has set itself the ambitious target of eliminating malaria by 2021. To continue tracking transmission to zero, new interventions, tools and approaches are required.

**Methods:**

Urban reactive case detection (RCD) was performed in Lusaka city from 2011 to 2015 to better understand the location and drivers of malaria transmission. Briefly, index cases were followed to their home and all consenting individuals living in the index house and nine proximal houses were tested with a malaria rapid diagnostic test and treated if positive. A brief survey was performed and for certain responses, a dried blood spot sample collected for genetic analysis. Aggregate health facility data, individual RCD response data and genetic results were analysed spatially and against environmental correlates.

**Results:**

Total number of malaria cases remained relatively constant, while the average age of incident cases and the proportion of incident cases reporting recent travel both increased. The estimated R_0_ in Lusaka was < 1 throughout the study period. RCD responses performed within 250 m of uninhabited/vacant land were associated with a higher probability of identifying additional infections.

**Conclusions:**

Evidence suggests that the majority of malaria infections are imported from outside Lusaka. However there remains some level of local transmission occurring on the periphery of urban settlements, namely in the wet season. Unfortunately, due to the higher-than-expected complexity of infections and the small number of samples tested, genetic analysis was unable to identify any meaningful trends in the data.

## Background

Transmitted by mosquitoes of the *Anopheles* genus, malaria killed an estimated one million people in the year 2000, the vast majority of whom lived in sub-Saharan Africa. Scale-up of insecticide-treated mosquito nets (ITN) to prevent malaria transmission and effective artemisinin-based combination therapy (ACT) to treat malaria among those infected have greatly reduced the burden of malaria in sub-Saharan Africa [1]. Inspired by the progress that these interventions have made, attention is turning away from just controlling malaria disease and reducing deaths to eliminating transmission of the parasite. Zambia is one country that has made great strides in reducing malaria through implementing proven interventions [2], and has now set itself the ambitious goal of eliminating malaria by 2021 [3]. To realize this goal, elimination will have to be achieved in all environmental settings.

Malaria transmission is most intense in rural areas [4, 5], which provide preferred habitat for the *Anopheles* mosquitoes that tend to lay eggs in clean water. While rural settings contribute the majority of transmission events, urban malaria persists and unless understood presents a threat to elimination [4, 6]. Many malaria-endemic regions are becoming more urban [7]. Indeed Zambia’s urban population increased from 35% of the total in 2000 to 40% in 2010 [8].

In Zambia, like many developing countries, urban growth is fastest in unplanned settlements that are prone to flooding [9], and hence may be at increased risk for malaria transmission. Unplanned peri-urban areas lack sewers or drainage systems, which allows for pooling of water to provide breeding sites for malaria vectors and increase the risk of malaria transmission [10–15]. Furthermore, unplanned settlements are typically built upon less desirable land, and often in proximity to existing natural breeding sites such as swamps or other hydrological networks associated with increased malaria risk [11, 13, 16–19]. Finally, agricultural activities, with their associated irrigation systems, can provide breeding sites and, therefore, increase malaria transmission in urban and peri-urban areas [19–23]. The spatial heterogeneity of these factors in urban areas leads to huge variation in the entomological inoculation rate (EIR) both across and within urban cities throughout malaria endemic regions. Keiser et al. found the EIR varied from 0 to 54 infective bites per person per year in urban areas throughout sub-Saharan Africa [24], although this range was estimated before the escalation of ITNs and ACT across the continent [25].

In Lusaka, the majority of malaria cases are associated with travel outside the city [26]. Because of the lower inherent transmission capacity for urban malaria, travel outside of urban areas to areas of higher malaria transmission is a primary risk factor for a case [27–30]. The risk that a traveller who acquires a malaria infection poses to neighbors upon the traveller’s return, i.e. the risk of onward transmission, depends upon the transmission capacity of the traveller’s return site [31], and will vary based upon the characteristics described previously.

In most settings it is possible to measure and then track changes in malaria transmission dynamics through cross-sectional parasite prevalence surveys, longitudinal routine health system metrics, or longitudinal entomological surveillance [32]. However, as malaria approaches elimination, the ability to define transmission with statistical confidence requires sample sizes that are often unachievable due to the rarity of malaria infections. While the rarity of an event cannot be changed, in this case the presence of a parasite in a person, the ability to sensitively and specifically detect and the amount of information derived from those events can be augmented through molecular tools. For example, PCR can be used to identify infections that are below the level of detection of rapid diagnostic tests (RDT) or microscopy. Furthermore, genetic analysis can determine both the complexity of infection i.e. the number of malaria co-infections, and/or the genetic haplotype of the infections. These genetic analyses have been used to show changes in transmission [33], as well as clonal expansion during an epidemic [34]. Where possible, molecular work, as reported here, should be performed locally [35] to ensure data is understood and applied to decision-making.

This manuscript examines the central question of whether there is ongoing malaria transmission within urban Lusaka, Zambia. Further, the ability to combine molecular and spatial tools to first identify whether transmission is occurring, and second to identify areas where transmission is more likely to be occurring, was examined.

## Methods

### Study site

**Fig. 1:**
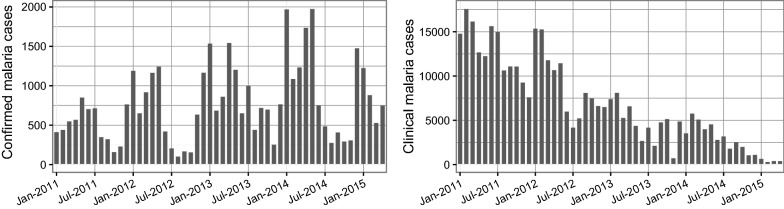
Trends in laboratory-confirmed and unconfirmed (clinical) malaria cases in the health management information system (HMIS) for all government health facilities in Lusaka district, Zambia

**Fig. 2:**
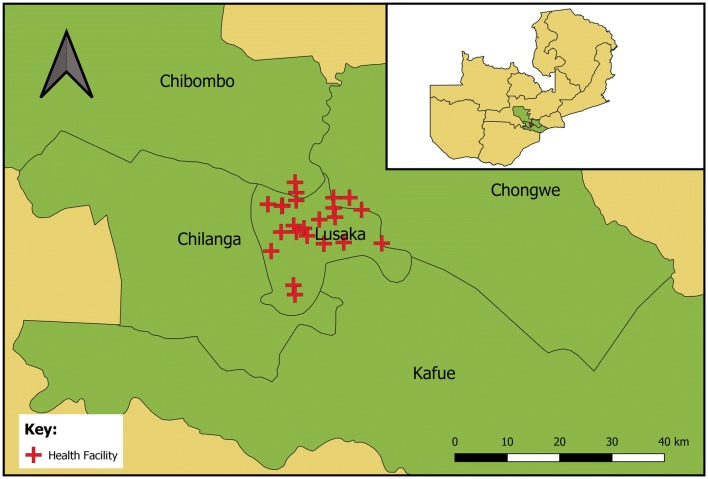
Location of 27 health facilities enrolled in this study (red cross) and names of districts. Inset map shows outline of Zambia by province with Lusaka district (red) and surrounding districts of Lusaka Province (Chilanga, Kafue, Chongwe and Chibombo, green)

Lusaka is the capital of Zambia and the prime economic hub of the country. The city lies at ~ 1300 m above sea level, and although it does not snow, temperatures in the cold season can drop to below 10 °C. Like many major cities in lower-income countries, Lusaka is made up of a combination of planned and unplanned settlements, and its workforce includes both formal and informal occupations. No malaria infections have been found within Lusaka in any of the Malaria Indicator Surveys since 2006, yet cases continue to be found through the public health system (Fig. 1). The reported primary malaria vectors in Lusaka are *Anopheles gambiae* sensu stricto (s.s.) and *Anopheles arabiensis* [36], however entomological surveillance has been challenging due to the very low vector numbers. Malaria testing and treatment is free to all individuals in the public health centres, which have improved their malaria case management dramatically in recent years [26]. A total of 27 health facilities within the city of Lusaka and under the management of the Lusaka District Health Management Team (80% of total health facilities) were included in this study (Fig. 2). Ten of these facilities were included throughout the 2011–2015 period, while 17 additional facilities were enrolled only during the final year. Facilities without an environmental health technician (EHT) were not included as the EHT co-ordinated the RCD responses.

### Reactive case detection

With funding from the Presidents Malaria Initiative (PMI), reactive case detection (RCD) commenced in 10 health facilities in Lusaka in 2011 with the goal of improving understanding of malaria transmission within the city and finding problematic transmission hotspots [37]. Prior to this date, no community follow-ups had been made for any health facility (HF) incident case. RCD has been described in detail elsewhere [37]. In brief, a team follows up a confirmed index case and tests the index case household and 9 closest neighbouring houses for malaria infection, treating those who test positive.

While almost all government health facilities in Lusaka participated at some point in intensified surveillance activities, human and financial resources were insufficient to follow up all HF incident cases, particularly in the wet season. For those index cases followed-up, a range of data was collected for both index cases and RCD participants, including demographic details, symptoms, travel, and malaria infection history in the last month (see Additional files 1 and 2). Household level data, including GPS location and history of IRS in the last 12 months, were also collected. In 2014, a grant from the Malaria Eradication Scientific Alliance (MESA), allowed RCD operations to be intensified and expanded to a total of 27 health facilities in the city. The expansion ensured more case investigations were performed and the collection of dried blood spots (DBS) from consenting individuals was added to the information previously collected.

### Data sources

Herein the RCD database, which was collected from 10 HFs in 2011–2014, and 27 HFs in 2014–2015 was utilized. The database contains more information than the standard health management information system in Zambia, with numerous characteristics about each incident malaria case including age, travel history, sex, and whether the case was followed up through RCD. The database also includes information of RCD responses, with individual-level information for all RCD households and participants.

### Descriptive analysis

From the RCD data, trends were analysed as follows. First, the proportion of incident malaria cases that reported travel outside Lusaka district in the previous 1 month was determined. Assuming that at least some portion of incident cases reporting travel outside Lusaka were imported, the formula from Churcher et al. was used to estimate a crude reproductive number (R_0_) for the city [38]. The tolerance of the importation and travel assumption was tested by running simulations of different levels of cases reporting travel outside Lusaka being considered as imported. Second, the age of incident malaria cases over time was determined, as the mean age of incident malaria in a population is an indicator of the intensity of malaria transmission [39–41].

### Environmental analysis

The topographical position index (TPI) and topographical wetness index (TWI) are associated with increased risk of malaria vector breeding sites and in some cases increased risk of malaria transmission [42–45]. Both indices are derived from a digital elevation model. Google Earth Engine was used to calculate TWI as well as TPI at resolutions of 300 m and 2000 m. Additionally, enhanced vegetation index at a spatial scale of 250 m and monthly temporal scale were retrieved. Using Quantum GIS version 2.0.1 and the OpenLayers plugin, uninhabited areas, defined as an area without a rooftop, were traced from satellite imagery. The Euclidean distance from the geocoordinates of the index case household to the nearest uninhabited area in increments of 50 ms, i.e. 0–50 m, 50–100 m, were then measured. These environmental measures were matched to the geo-coordinates of RCD participant households in 2014 and 2015 using the Raster package [46, 47], in R version 3.3.1 [48].

### Regression model analysis

Two separate outcomes with regards to the RCD data was examined. First, the probability of testing positive for a *P. falciparum* infection during RCD with RCD participants as the unit of analysis was assessed, and second the probability of finding a *P. falciparum* infection during RCD with each RCD investigation as the unity of analysis.

Factors associated with testing positive for a *P. falciparum* infection during RCD were examined as follows. A priori hypotheses suggested that travel outside of Lusaka, season, person’s age, person’s gender, and location of the household (person living in the index case household or not) could be associated with having a *P. falciparum* infection. A logistic regression approach with the index case included as a random intercept in the model was utilized. The general model used to assess the relationship between testing positive for a *P. falciparum* infection during RCD and the hypothesized factors is given by the following equations:

$$y_{ijk} |\pi_{ijk} \sim Binomial\left( {1,\pi_{ijk} } \right)$$

$$logit\left( {\pi_{ijk} } \right) = \beta_{1} Travel_{i} + \beta_{2} Season_{k} + \beta_{3} Age_{i} + \beta_{4} Sex_{i} + \beta_{5} Location_{j} + \chi_{k}$$

$$\chi_{k} \sim N\left( {0,\delta } \right)$$

where π_ijk_ is a dichotomous outcome for person *i* in household *j* participating in the RCD for index case *k*; Travel_i_ is whether that person travelled outside Lusaka in the previous 2 weeks or not; Season_k_ is whether the RCD was conducted during the high transmission season or not; Age_i_ is the age of the person categorized as < 5, 5–15, and > 15 years of age; Sex_i_ is whether the person is male or female; Location_j_ is whether the person lives in the index case household or not; and χ_k_ is a random intercept for RCD activities associated with index case *k* that is assumed to be normally distributed with a mean of zero.

Factors associated with finding an RDT-positive individual during RCD were examined as follows. A priori hypotheses suggested that travel outside of Lusaka, season, distance from uninhabited areas, age, and sex (gender) of the incident malaria case could be associated with finding more positives during RCD. In the wet season distance from uninhabited area, travel, season, age and gender were all retrieved from the surveillance database. The number of positives found during an RCD response were skewed right and overdispersed, so a negative-binomial regression was used to determine the association with the hypothesized factors. The general model used to assess the relationship between testing positive for a *P. falciparum* infection during RCD and the hypothesized factors is given by the following equations:

$$\mu_{i} = e^{{ln\left( {t_{i} } \right) + \beta_{1} Location_{i} \times \beta_{2} Season_{i} + \beta_{3} Age_{i} + \beta_{4} Sex_{i} + \beta_{5} Travel_{i} }}$$

where μ_I_ is the number of *P. falciparum* infections found during RCD of index case *i*; *t*_*i*_ is the number of people tested during RCD of index case *i*; *Location*_*i*_ is whether the household was located within 250 metres of an uninhabited area of Lusaka or not; *Season*_*i*_ is whether the RCD was conducted during the high or low malaria transmission season; *Age*_*i*_ is the age of the index case categorized as < 5, 5–15, or > 15 years; *Sex*_*i*_ is whether the index case was male or female; and *Travel*_*i*_ is whether or not the index case travelled outside Lusaka in the previous 2 weeks.

All regression analyses were conducted in Stata version 13.1.

### Genetic analysis

From 2014, a further aim of collecting a DBS from every index case and all RCD participants was added to the protocol. Unfortunately, challenges in the field meant that some DBS were not collected, were incorrectly labelled, incorrectly stored or lost during transit to the laboratory. A subset of RCD responses were selected for molecular analysis based on the completeness of the sample record, i.e. only those responses with a complete or near-complete (> 85%) DBS sample repository were analysed.

DNA was extracted from RDT negative DBS either individually or in pools of 10 using a QIAamp (Qiagen) mini-spin column or DNA IQ system (Promega) as per manufacturer’s instructions, and amplified using photo-induced electron transfer PCR (PET-PCR) as previously described [49]. Positive pools were deconvoluted to identify individual positives. PCR/RDT positives were then genotyped/barcoded using the Taqman assay as described elsewhere [50].

Barcoded samples with ≥ 11 missing loci (out of 24), were classified as incomplete and removed from any further analysis. Infections were classified as polyclonal using a cutoff of ≥ 4 loci with a mixed infection call [33, 50, 51]. Genetic relatedness was calculated using a modified SNP π [52], which accounts for samples with missing data and mixed infections [52], and visualized using a neighbour joining phylogenetic tree.

The complexity of infection (COI) was determined for complete barcoded samples using the COI Likelihood (COIL) calculator developed by Galinsky et al. [53]. In brief, COIL uses Bayesian methodology to estimate the probable number of infections that are present within a single sample, based on the number of isolated pairs.

## Results

### Trends in incident malaria cases 2011–2015

**Fig. 3:**
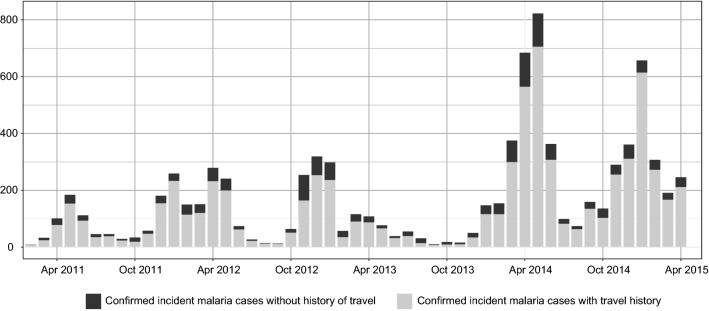
Trends in reactive case detection system in Lusaka, Zambia showing confirmed incident malaria cases with and without a history of travel in the last month

**Fig. 4:**
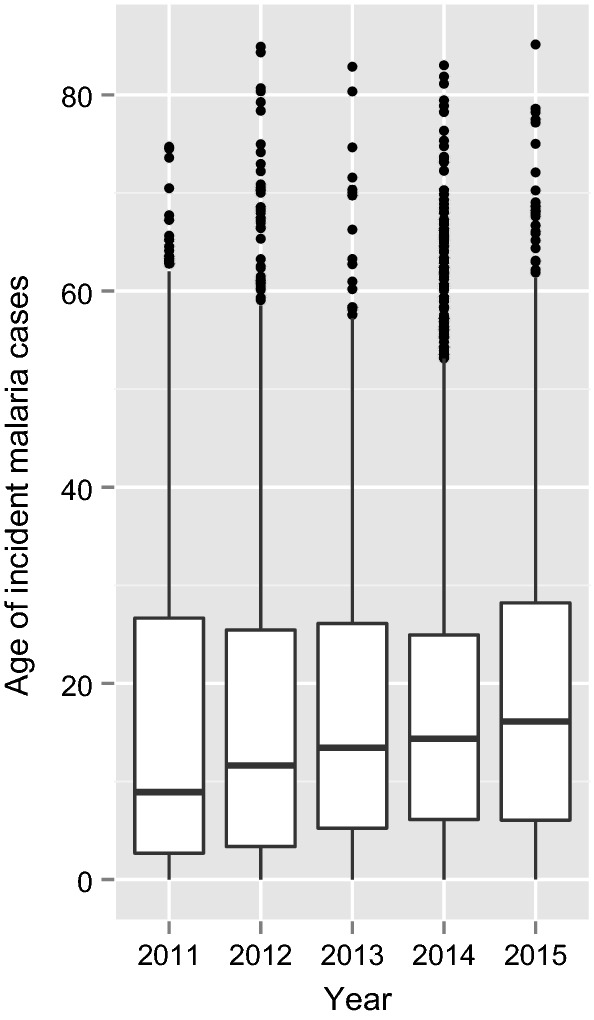
Age distribution of RCD incident malaria cases in Lusaka, Zambia. Box represents interquartile range, dots represent statistical outliers > 2 standard deviations above the mean

**Fig. 5:**
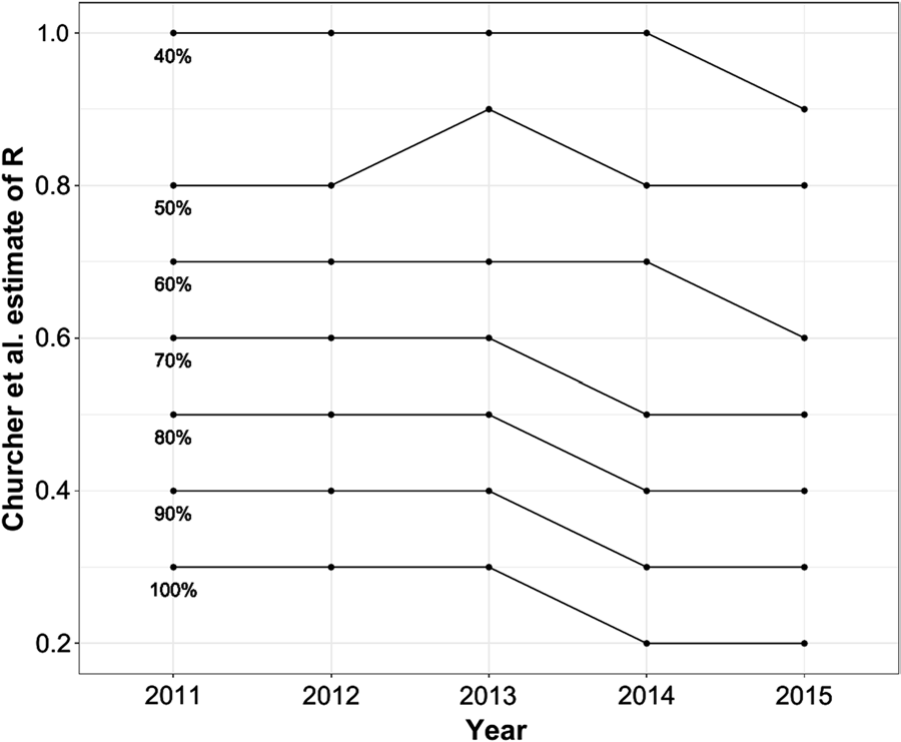
R_0_ estimated from enhanced surveillance system, with varying levels, from 40 to 100%, of assumed importation for incident malaria cases reporting history of travel within the last month

Between 2011 and 2015, 14,966 confirmed incident malaria cases were reported for all health facilities within Lusaka district, of which 8723 confirmed cases were reported from health facilities which were participating in this study at the time (10 HFs in 2011–2014, 27 HFs in 2014–2015). Among these confirmed incident malaria cases the majority reported travel outside Lusaka in the previous 2 weeks (Fig. 3, Table 1), and the median age of incident malaria cases steadily increased from 8.9 years of age in 2011 (interquartile range = 2.7–26.7) to 16.1 years of age in 2015 (interquartile range = 6.1–28.2) (Fig. 4). Assuming that at least 40% of incident cases reporting travel outside Lusaka are imported cases, Lusaka has an estimated R_0_ < 1 since 2011, with a decrease in 2014–2015 compared to 2011–2013 (Fig. 5).

**Table 1:** Descriptive characteristics of incident malaria cases in Lusaka 2011–2015

Year	Number of health facilities participating	Confirmed incident malaria cases	Number of cases reporting travel (%)	Median age in years (inter-quartile range)
2011	5	855	694 (81%)	9 (3**–**27)
2012	20	1869	1497 (81%)	12 (3**–**25)
2013	22	899	684 (79%)	13 (5**–**26)
2014	25	3688	3080 (86%)	14 (6**–**25)
2015	19	1411	1273 (90%)	16 (6**–**28)

### Factors associated with finding additional positives during case investigations

**Fig. 6:**
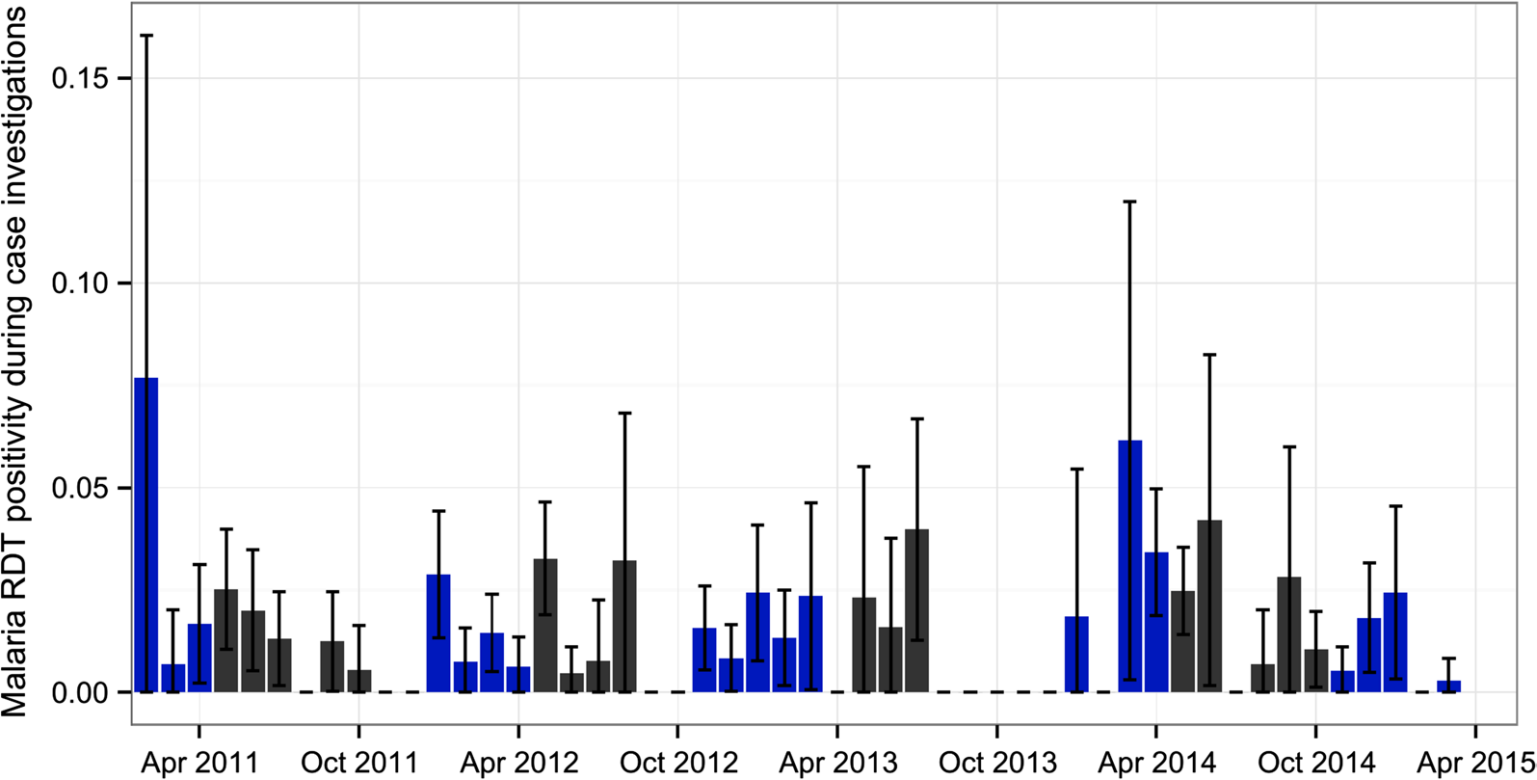
Distribution of malaria test positivity with 95% confidence intervals indicated by error bars among RCD participants from 2011–2015. Wet season (blue) and dry season (black) are indicated

From a total of 8723 confirmed incident malaria cases, 428 (4.9%) index malaria cases were investigated during RCD, enrolling 11,954 RCD participants (community members tested by the RCD system), and 206 RDT-positive malaria infections found (RCD incident cases). Test positivity during RCD was typically lower than 5% (mean 1.71% ± SD 1.65%), with higher test positivity during the high transmission season compared to the low transmission season (Fig. 6).

Among the RCD participants, a number of factors were associated with increased odds of individuals testing positive including seasonality and distance to uninhabited areas, travel outside of Lusaka in the past month, age (children aged 5–15), and living in the same household as the index case (Table 2). Additionally, the proximity of the index case household to uninhabited areas of Lusaka during the high transmission season was associated with an increased probability of finding malaria infections during RCD (Table 3). However, no association between RDT-positive RCD participants and any index case demographics, including travel history (Table 3) was detected. In addition, no association was found between finding a positive during RCD and any of the remotely sensed environmental factors that were examined, specifically TPI at scales of 300 m and 2000 m, TWI, mean EVI, monthly EVI, minimum EVI, maximum EVI and altitude.

**Table 2:** Random effects logistic regression results assessing, among RCD participants, the association between risk factors and testing positive for a malaria infection during a case investigation

Variable	Factor	Unadjusted IRR (95% CI)	*P* value	Adjusted IRR (95% CI)	P-value
Season and distance from uninhabited areas	Dry season > 250 m	Reference	Reference	Reference	Reference
	Dry season and ≤ 250 m	1.88 (0.80–4.42)	0.150	3.74 (0.50–28.13)	0.200
	Wet season and > 250 m	2.06 (0.85–5.03)	0.150	6.16 (0.76–49.77)	0.088
	Wet season and ≤ 250 m	2.27 (1.01–5.09)	0.048	8.90 (1.31–60.49)	0.025
Age of person tested	< 5 years	Reference	Reference	Reference	Reference
	5–15 years	1.74 (1.27–2.39)	0.001	2.01 (1.29–3.11)	0.002
	> 15 years	1.24 (0.85–1.81)	0.273	1.47 (0.87–2.46)	0.146
Sex of person tested	Female	Reference	Reference	Reference	Reference
	Male	1.11 (0.83–1.49)	0.477	1.10 (0.74–1.64)	0.639
Travel in past 1 month	None	Reference	Reference	Reference	Reference
	Outside Lusaka	11.06 (5.14–23.80)	< 0.0001	13.65 (6.28–29.64)	< 0.0001
Location of household	Adjacent to index case	Reference	Reference	Reference	Reference
	Index case house	4.33 (3.18–5.90)	< 0.0001	4.03 (2.57–6.33)	< 0.0001

A total of 11,954 individuals were tested for malaria during 428 case investigations

**Table 3:** Negative binomial regression analysis assessing the association between risk factors and the number of RDT-positive individuals found among reactive case detection participants

Variable	Factor	Unadjusted IRR (95% CI)	P-value	Adjusted IRR (95% CI)	P-value
Season and distance from uninhabited areas	Dry season > 250 m	Reference	Reference	Reference	Reference
	Dry season and ≤ 250 m	1.77 (0.79–3.94)	0.119	1.82 (0.81–4.10)	0.147
	Wet season and > 250 m	1.95 (0.84–4.54)	0.164	2.06 (0.88–4.81)	0.096
	Wet season and ≤ 250 m	2.06 (0.97–4.39)	0.061	2.14 (0.99–4.59)	0.051
Age of index case	< 5 years	Reference	Reference	Reference	Reference
	5–15 years	1.20 (0.76–1.88)	0.432	1.20 (0.76–1.89)	0.441
	> 15 years	1.36 (0.88–2.11)	0.164	1.37 (0.88–2.12)	0.163
Sex of index case	Female	Reference	Reference	Reference	Reference
	Male	1.22 (0.85–1.76)	0.287	1.20 (0.83–1.74)	0.342
Index case travelled in the past month	None	Reference	Reference	Reference	Reference
	Outside Lusaka	0.81 (0.48–1.37)	0.437	0.76 (0.44–1.29)	0.302

N = 428 case investigations

### Genetic analysis

A subset of samples from 65 reactive case detection responses comprising 645 people (65 index cases and 580 household members) from 204 houses were further assessed by genetic analysis. The RDT positivity during the responses was 0.47% (3/580), while 446 individuals had DBS collected (59 index cases and 387 household members). PCR analysis identified two false positives (1 index case and 1 household member), and 4 false negative RDTs (all household members). The latter increased the positivity rate, to 1.55% (6/387), an approximate threefold increase.

### Barcoding

**Fig. 7:**
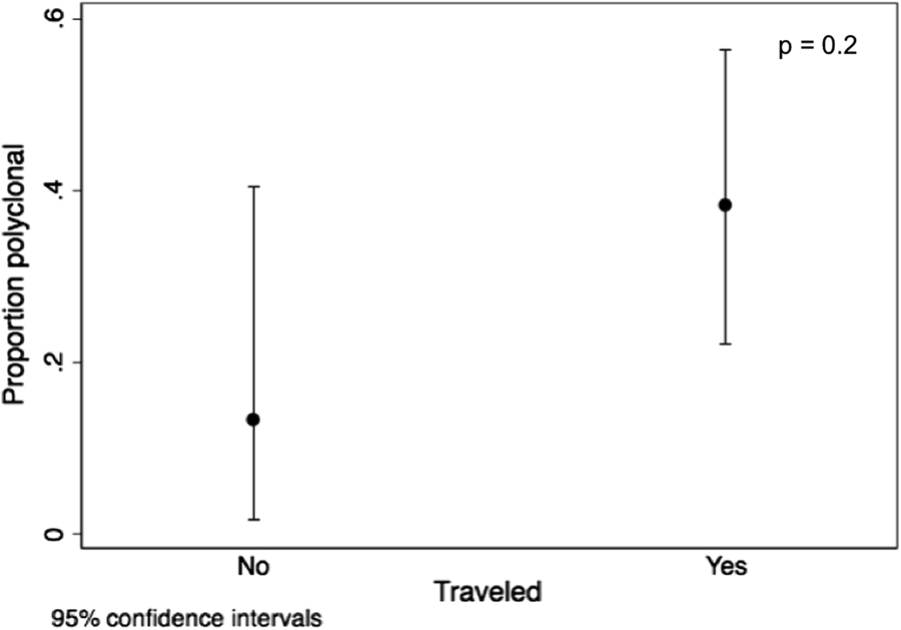
Proportion of polyclonal infections in malaria-positive RCD participants with and without a travel history

**Fig. 8:**
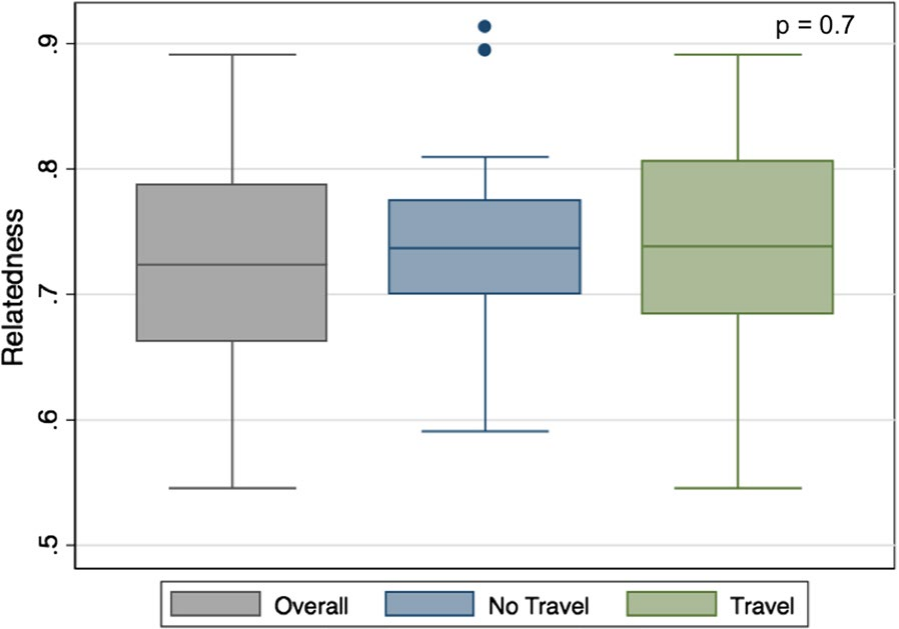
Overall genetic relatedness of parasites found during RCD, comparing RCD participants with and without a travel history

Positive molecular barcode data was generated from 72 individuals, with a range of completeness. Of these, 22 samples had ≥ 11 missing loci (out of 24), were classified as incomplete and removed from any further analysis. The remaining 50 individuals had a median age of 17 years and had a high proportion of travel (67%) with a median travel time of 8.5 days. The proportion of polyclonal infections was moderate (28%) and genetic relatedness was high (72%) (Additional file 3: Table S1). Phylogenetic analysis did not show any evidence of clustering of genetic structure between individuals with or without travel history. Individuals with a travel history were slightly older (18 vs. 10 years old, p = 0.5), more likely to be an index case (97% vs. 87%, p < 0.05), have more polyclonal infections (38% vs. 13%, p = 0.2), more febrile (82% vs. 73%, p = 0.2), and have slightly less related infections (74% vs. 75%, p = 0.7) in comparison to individuals who did not travel (Additional file 3: Table S1, Figs. 7 and 8). Individuals with a travel history had a slightly higher genetic relatedness compared to the overall genetic relatedness of individuals without a travel history (Fig. 8).

### COIL analysis

COIL analysis predicted that 54 of 71 (76%) genotyped malaria samples were from single infections, however 19 of these predicted single infections had posterior probabilities < 0.80 due to missing SNPs. Of the 50 genotyped samples with high statistical certainty, 32 were single infections (64%), 12 were dual infections (24%) and six had three or more infections (12%).

## Discussion

In this study, information from incident case information, particularly spatial location, and later with genetic analyses of malaria infections was used to spatially review the extent and location of malaria transmission in the city of Lusaka, Zambia, an area approaching malaria elimination. The rationale for such an approach was to enhance the signal to noise ratio by swapping a binary uninfected/infected output to an exponentially richer haplotype/multiplicity of infection (MOI) output. Combined with the spatial data from reactive case detection, it was hoped that relationships between individual infections, through haplotype matching/relatedness, could be identified as well as estimates of transmission determined from the MOI. However, the study was not powered to measure a specific deviation, but rather aimed to describe the parasite population from a molecular point of view.

### Transmission trends

While the total number of incident malaria cases was relatively constant, evidence was found to support the conclusion that transmission decreased over the study period from the standard measures of transmission available in the HMIS. This included an increase in the median age of incident cases, as well as a declining R_0_ of < 1, over time. Interestingly, the probability of finding infections during RCD was increased if the index malaria case lived on the periphery of human settlement and the index case was reported during the wet season, but not the dry season. Given these results from RCD, it appears that there may be malaria transmission which is not associated with travel occurring along the periphery of human settlements during the wet season. A key challenge for RCD is determining the appropriate range for a response. In this study, living in the index house was associated with testing positive (adjusted IRR 4.03, Table 2), however a large number of positives were identified in non-index houses. Without additional information on the relationship between, or source of these different infections, it is unclear whether the radius used in this study, nine closest neighboring houses, is sufficient or overkill.

Unfortunately, it was not possible to demonstrate any associations between remotely sensed topographical information and the probability of finding a malaria infection during RCD, other than distance from uninhabited areas. RDT-diagnosed malaria infections were used as the primary outcome in these analyses, which could have added noise through false positives and false negatives. While this noise is likely to be present, it is unlikely to entirely account for the null results observed. More research on urban malaria transmission dynamics is needed, particularly around risk mapping in urban environments. Distance to uninhabited areas is a known factor that increases the probability of being at risk of malaria transmission [54, 55], but more research is needed to identify specific characteristics of areas that make them more probable to continue onward malaria transmission so that they can be either modified or used to target for malaria control.

As Lusaka aims to become free of malaria transmission, increased mosquito control in the periphery may be of benefit, and linking malaria surveillance to vector control microplanning processes is important. The results found herein suggest that there is vectoral capacity in the periphery to facilitate malaria transmission if malaria parasites are present or imported. Malaria control programmes aiming for elimination may be more successful when focusing on locations of human settlements where sufficient habitat enables malaria transmission [56].

Although of benefit in describing malaria trends [57], human movement is more challenging to address as a driver of malaria transmission and in this study was not associated with finding malaria cases among RCD participants. In our view, chemoprophylaxis for travellers, while potentially challenging to implement would help alleviate malaria cases, but may not reduce transmission in the city.

### Genetics

In contrast to the evidence derived from HMIS and RCD data, it was much harder to draw a clear conclusion from the spatial genetics data. Firstly, in this study, MOI (as determined by COIL) was higher than expected, even in individuals with no history of travel. While MOI is thought to correlate directly with transmission, where a large proportion of the infections are imported this relationship may be skewed. For example, if, as suggested here, local transmission represents a relatively small, but persistent fraction of the source of infections, the high importation of diverse polymorphic infections likely sustains a high MOI for any locally transmitted cases. Where local transmission chains are very short, as supported by estimates of R reported here, this artificially high MOI would be more pronounced. Interestingly, the proportion of polygenomic infections correlated with travel (Fig. 7) suggesting that MOI decreases with local transmission.

The ability to utilize SNP-barcode methods to successfully identify individual haplotypes decreases with increasing levels of MOI, making matching/determining relatedness between individual infections much harder. Indeed, it is possible that identical haplotypes were not identified as they were masked by other haplotypes in individuals with more than one parasite present. When designing the study it was hoped that genetic relatedness correlated across space at a local spatial scale (in terms of metres) for individuals without any recent travel history [58]. However, due to the high MOI and high importation, with the majority of infections likely acquired across a large area of Zambia, it was not possible to perform genetic spatial analyses with the SNP-barcode methods. Future work aiming to examine spatial genetics as potential tools for assessing the epidemiology of malaria parasites should consider genetic barcoding methods such as amplicon deep sequencing which may allow analysis of polyclonal samples [59]. Otherwise, researchers should seek areas of low transmission where imported cases are relatively few. It is reasonable to expect the ability to differentiate imported from locally-acquired infections to increase in resolution as the repository of Zambian barcodes grows. Future analyses of these data when equipped with a better understanding of the identity and spatial distribution of Zambian parasite populations may yield clearer results.

## Conclusions

Results suggest there may be two separate malaria transmission phenomena occurring simultaneously in Lusaka: low-level transmission circulating in the periphery as well as a high number of imported malaria cases. The vast majority (> 90%) of malaria cases is likely a result of travel outside Lusaka, however there appears to be persistent unrelated malaria transmission on the periphery of the city. Spatial analyses can be combined with genetic analyses to investigate infectious diseases, but may be limited in their findings due to the rarity of the infection and are further complicated by infections with a multiplicity greater than one. The macro-level tools of median age of malaria cases and Churcher’s formula are useful in describing the former, however they appear less useful in describing the latter. As Zambia continues its path towards malaria elimination, further fine-scale surveillance data must be collected to better understand urban and peri-urban transmission dynamics and to plan, coordinate and monitor malaria interventions.

## Supplementary information

**Additional file 1.** Household data collection form for individuals enrolled in reactive case detection.

**Additional file 2.** Index data collection form for individual diagnostically confirmed to have malaria at a health facility.

**Additional file 3: Table S1.** Characteristics of incident and RCD positive individuals with and without a travel history.

## Data Availability

Data collected for this study may be obtained through written request to the corresponding author, subject to approval from the Zambian Ministry of Health.

## Abbreviations

ACT: artemisinin-based combination therapy
EIR: entomological inoculation rate
ITN: insecticide-treated mosquito net

## References

[1] Bhatt S, Weiss DJ, Cameron E, Bisanzio D, Mappin B, Dalrymple U (2015). The effect of malaria control on *Plasmodium falciparum* in Africa between 2000 and 2015. Nature.

[2] Chizema-Kawesha E, Miller JM, Steketee RW, Mukonka VM, Mukuka C, Mohamed AD (2010). Scaling up malaria control in Zambia: progress and impact 2005–2008. Am J Trop Med Hyg.

[3] Ministry of Health, National Malaria Elimination Centre. National Malaria Elimination Strategic Plan 2017–2021. Lusaka, Zambia; 2015.

[4] Robert V, Macintyre K, Keating J, Trape J-FF, Duchemin J-BB, Warren M (2003). Malaria transmission in urban sub-Saharan Africa. Am J Trop Med Hyg.

[5] Hay SI, Guerra CA, Tatem AJ, Atkinson PM, Snow RW (2005). Urbanization, malaria transmission and disease burden in Africa. Nat Rev Microbiol.

[6] Wilson ML, Krogstad DJ, Arinaitwe E, Arevalo-Herrera M, Chery L, Ferreira MU (2015). Urban malaria: understanding its epidemiology, ecology, and transmission across seven diverse ICEMR network sites. Am J Trop Med Hyg.

[7] Tatem AJ, Gething PW, Smith DL, Hay SI (2013). Urbanization and the global malaria recession. Malar J..

[8] Central Statistic Office [Zambia], Ministry of Health [Zambia], ICF International. Zambia Demographic and Health Survey 2013–14. Rockville, Maryland, USA; 2014.

[9] Nchito WS (2007). Flood risk in unplanned settlements in Lusaka. Environ Urban..

[10] Afrane YA, Klinkenberg E, Drechsel P, Owusu-Daaku K, Garms R, Kruppa T (2004). Does irrigated urban agriculture influence the transmission of malaria in the city of Kumasi, Ghana?. Acta Trop.

[11] Baragatti M, Fournet F, Henry MC, Assi S, Ouedraogo H, Rogier C (2009). Social and environmental malaria risk factors in urban areas of Ouagadougou, Burkina Faso. Malar J..

[12] Ferrari G, Ntuku HM, Schmidlin S, Diboulo E, Tshefu AK, Lengeler C (2016). A malaria risk map of Kinshasa, Democratic Republic of Congo. Malar J..

[13] Kabaria CW, Molteni F, Mandike R, Chacky F, Noor AM, Snow RW (2016). Mapping intra-urban malaria risk using high resolution satellite imagery: a case study of Dar es Salaam. Int J Health Geogr..

[14] Alemu A, Tsegaye W, Golassa L, Abebe G (2011). Urban malaria and associated risk factors in Jimma town, south-west Ethiopia. Malar J..

[15] de Castro MC, Sawyer DO, Singer BH (2007). Spatial patterns of malaria in the Amazon: implications for surveillance and targeted interventions. Health Place..

[16] Akono PN, Mbida JAM, Tonga C, Belong P, Ngo Hondt OE, Magne GT (2015). Impact of vegetable crop agriculture on anopheline agressivity and malaria transmission in urban and less urbanized settings of the South region of Cameroon. Parasit Vectors..

[17] Clark TD, Greenhouse B, Njama-Meya D, Nzarubara B, Maiteki-Sebuguzi C, Staedke SG (2008). Factors determining the heterogeneity of malaria incidence in children in Kampala, Uganda. J Infect Dis.

[18] Ferrão JL, Mendes JM, Painho M, João SZ (2016). Spatio-temporal variation and socio-demographic characters of malaria in Chimoio municipality, Mozambique. Malar J..

[19] Dongus S, Nyika D, Kannady K, Mtasiwa D, Mshinda H, Gosoniu L (2009). Urban agriculture and Anopheles habitats in Dar es Salaam, Tanzania. Geospat Health..

[20] Klinkenberg E, McCall P, Wilson MD, Amerasinghe FP, Donnelly MJ (2008). Impact of urban agriculture on malaria vectors in Accra, Ghana. Malar J..

[21] Matthys B, N’Goran EK, Koné M, Koudou BG, Vounatsou P, Cissé G (2006). Urban agricultural land use and characterization of mosquito larval habitats in a medium-sized town of Côte d’Ivoire. J Vector Ecol..

[22] Matthys B, Koudou BG, N’Goran EK, Vounatsou P, Gosoniu L, Kone M (2010). Spatial dispersion and characterisation of mosquito breeding habitats in urban vegetable-production areas of Abidjan, Côte d’Ivoire. Ann Trop Med Parasitol.

[23] Yadouléton A, N’Guessan R, Allagbé H, Asidi A, Boko M, Osse R (2010). The impact of the expansion of urban vegetable farming on malaria transmission in major cities of Benin. Parasit Vectors..

[24] Keiser J, Utzinger J, de Castro M, Smith TA, Tanner M, Singer BH (2004). Urbanization in sub-saharan Africa and implication for malaria control. Am J Trop Med Hyg.

[25] Bhatt S, Weiss DJ, Mappin B, Dalrymple U, Cameron E, Bisanzio D (2015). Coverage and system efficiencies of insecticide-treated nets in Africa from 2000 to 2017. Elife..

[26] Chisha Z, Larsen DA, Burns M, Miller JM, Chirwa J, Mbwili C (2015). Enhanced surveillance and data feedback loop associated with improved malaria data in Lusaka, Zambia. Malar J..

[27] Diallo A, Ndam NT, Moussiliou A, Santos S, Ndonky A, Borderon M (2012). Asymptomatic carriage of plasmodium in urban Dakar: the risk of malaria should not be underestimated. PLoS One.

[28] Domarle O, Razakandrainibe R, Rakotomalala E, Jolivet L, Randremanana RV, Rakotomanana F (2006). Seroprevalence of malaria in inhabitants of the urban zone of Antananarivo, Madagascar. Malar J..

[29] Osorio L, Todd J, Pearce R, Bradley DJ (2007). The role of imported cases in the epidemiology of urban Plasmodium falciparum malaria in Quibdó, Colombia. Trop Med Int Health..

[30] Siri JG, Wilson ML, Murray S, Rosen DH, Vulule JM, Slutsker L (2010). Significance of travel to rural areas as a risk factor for malarial anemia in an urban setting. Am J Trop Med Hyg.

[31] Le Menach A, Tatem AJ, Cohen JM, Hay SI, Randell H, Patil AP (2011). Travel risk, malaria importation and malaria transmission in Zanzibar. Sci Rep..

[32] Hay SI, Smith DL, Snow RW (2008). Measuring malaria endemicity from intense to interrupted transmission. Lancet Infect Dis..

[33] Daniels RF, Schaffner SF, Wenger EA, Proctor JL, Chang H-H, Wong W (2015). Modeling malaria genomics reveals transmission decline and rebound in Senegal. Proc Natl Acad Sci USA.

[34] Obaldia N, Baro NK, Calzada JE, Santamaria AM, Daniels R, Wong W (2015). Clonal Outbreak of *Plasmodium falciparum* Infection in Eastern Panama. J Infect Dis.

[35] Bridges DJ, Colborn J, Chan AST, Winters AM, Dengala D, Fornadel CM (2014). Modular laboratories - Cost-effective and sustainable infrastructure for resource-limited settings. Am J Trop Med Hyg.

[36] Chanda E, Baboo KS, Shinondo CJ (2012). Transmission attributes of periurban malaria in Lusaka, Zambia, precedent to the integrated vector management strategy: an entomological input. J Trop Med..

[37] Larsen DA, Chisha Z, Winters B, Mwanza M, Kamuliwo M, Mbwili C (2015). Malaria surveillance in low-transmission areas of Zambia using reactive case detection. Malar J..

[38] Churcher TS, Cohen JM, Novotny J, Ntshalintshali N, Kunene S, Cauchemez S (2014). Measuring the path toward malaria elimination. Science.

[39] Woolhouse MEJ (1998). Patterns in parasite epidemiology: the peak shift. Parasitol Today..

[40] O’Meara WP, Bejon P, Mwangi TW, Okiro EA, Peshu N, Snow RW (2008). Effect of a fall in malaria transmission on morbidity and mortality in Kilifi, Kenya. Lancet.

[41] Mogeni P, Williams TN, Fegan G, Nyundo C, Bauni E, Mwai K (2016). Age, spatial, and temporal variations in hospital admissions with malaria in Kilifi County, Kenya: a 25-year longitudinal observational study. PLoS Med..

[42] Bomblies A, Duchemin J-B, Eltahir EAB (2009). A mechanistic approach for accurate simulation of village scale malaria transmission. Malar J..

[43] Clennon JA, Kamanga A, Musapa M, Shiff C, Glass GE (2010). Identifying malaria vector breeding habitats with remote sensing data and terrain-based landscape indices in Zambia. Int J Health Geogr..

[44] Mushinzimana E, Munga S, Minakawa N, Li L, Feng C-C, Bian L (2006). Landscape determinants and remote sensing of anopheline mosquito larval habitats in the western Kenya highlands. Malar J..

[45] Nmor JC, Sunahara T, Goto K, Futami K, Sonye G, Akweywa P (2013). Topographic models for predicting malaria vector breeding habitats: potential tools for vector control managers. Parasit Vectors..

[46] Hijmans RJ, van Etten J. raster: Geographic analysis and modeling with raster data. R package version; 2012; Available from: http://cran.univ-lyon1.fr/web/packages/raster/.

[47] Hijmans RJ. Introduction to the’raster’package (version 2.0-08). 2012; http://probability.ca/cran/web/packages/raster/vignettes/Raster.pdf.

[48] R Core Development Team. R: A Language and Environment for Statistical Computing. http://wwwR-project.org/. R Foundation for Statistical Computing; 2010;.

[49] Lucchi NW, Narayanan J, Karell MA, Xayavong M, Kariuki S, DaSilva AJ (2013). Molecular diagnosis of malaria by photo-induced electron transfer fluorogenic primers: PET-PCR. PLoS ONE.

[50] Daniels R, Volkman SK, Milner DA, Mahesh N, Neafsey DE, Park DJ (2008). A general SNP-based molecular barcode for *Plasmodium falciparum* identification and tracking. Malar J..

[51] Daniels R, Chang H-H, Séne PD, Park DC, Neafsey DE, Schaffner SF (2013). Genetic surveillance detects both clonal and epidemic transmission of malaria following enhanced intervention in Senegal. PLoS One.

[52] Searle KM, Katowa B, Kobayashi T, Siame MNS, Mharakurwa S, Carpi G (2017). Distinct parasite populations infect individuals identified through passive and active case detection in a region of declining malaria transmission in southern Zambia. Malar J..

[53] Galinsky K, Valim C, Salmier A, de Thoisy B, Musset L, Legrand E (2015). COIL: a methodology for evaluating malarial complexity of infection using likelihood from single nucleotide polymorphism data. Malar J..

[54] Carter R, Mendis KN, Roberts D (2000). Spatial targeting of interventions against malaria. Bull World Health Organ.

[55] Bousema T, Griffin JT, Sauerwein RW, Smith DL, Churcher TS, Takken W (2012). Hitting hotspots: spatial targeting of malaria for control and elimination. PLoS Med..

[56] Larsen DA, Kangombe TN, Cheelo S, Hamainza B, Miller J, Winters A (2017). Location, location, location: environmental factors better predict malaria—positive individuals during reactive case detection than index case demographics in Southern Province, Zambia. Malar J..

[57] Wesolowski A, Eagle N, Tatem AJ, Smith DL, Noor AM, Snow RW (2012). Quantifying the impact of human mobility on malaria. Science.

[58] Guillot G, Estoup A, Mortier F, Cosson JF (2005). A spatial statistical model for landscape genetics. Genetics.

[59] Lerch A, Koepfli C, Hofmann NE, Messerli C, Wilcox S, Kattenberg JH (2017). Development of amplicon deep sequencing markers and data analysis pipeline for genotyping multi-clonal malaria infections. BMC Genomics..

