# Supplementary material for: The use of spatial and genetic tools to assess Plasmodium falciparum transmission in Lusaka, Zambia between 2011 and 2015
Source: Malar J. 2020 Jan 15;19:20. doi: 10.1186/s12936-020-3101-7 (PMC6964105; doi:10.1186/s12936-020-3101-7)
Supplement: Supplementary file 1 — Additional file 1. Household data collection form for individuals enrolled in reactive case detection. [file 12936_2020_3101_MOESM1_ESM.pdf]

Household form

HOUSE ______(1-10 for each response. Index house is #1)

Date * (dd/mm/yy)		Index Case Name (Surname, Firstname)		Responding Health Facility	

House Details							
Household Head (Surname, Firstname)		GPS co-ordinates (Left-hand corner from doorway of structure)	-_ _ . _ _ _ _ / _ _ . _ _ _ _ Latitude Longitude	Previous IRS (circle one)	Y / N / Unsure	Previous IRS date (# months ago)	

Household Member Details																							
Surname	Firstname	Date of Birth *	RDT Result (+ / -)	Symptoms (tick all that apply)								Travel History in last 1 month ONLY		Previous malaria in last 1 month ONLY		Treatment today							
				Fever	Headache	Cough	Diarrhoea	Vomiting	Problems breathing	Chest Pain	Other	Travel Length (days)	Place(s) visited outside of Lusaka	Diagnosis (RDT / Microscopy / Clinical)	Treatment taken	Pregnant?	Co-artem (pack size)				Other (list)	Refer to clinic?
																	6	12	18	24		

*please list all dates as Day/Month/Year e.g. 20/02/11 unless otherwise indicated

Interventions			
Number of nets distributed to household		Other recommendations	
