# Supplementary material for: The use of spatial and genetic tools to assess Plasmodium falciparum transmission in Lusaka, Zambia between 2011 and 2015
Source: Malar J. 2020 Jan 15;19:20. doi: 10.1186/s12936-020-3101-7 (PMC6964105; doi:10.1186/s12936-020-3101-7)
Supplement: Supplementary file 2 — Additional file 2. Index data collection form for individual diagnostically confirmed to have malaria at a health facility. [file 12936_2020_3101_MOESM2_ESM.pdf]

Index case form

Date *		Health Facility / Dept	

*please list all dates as Day/Month/Year e.g. 20/02/11

Patient Identity

Surname	First Name	Date of Birth *	Sex (circle one)
			M
			F

Diagnostics and Treatment

Symptoms (tick all that apply)

Fever	Headache	Cough	Diarrhoea	Vomiting	Problems breathing	Chest Pain	Other (specify)

Test Type (tick one)		Result (tick one)		Treatment provided today (tick one)							
				Coartem (pack size)				Fansidar	Quinine Tablets	Quinine Injection	Other (specify)
RDT		Positive		6	12	18	24				
Microscopy		Negative									

Travel History (in last 1 month)

Has this person travelled outside Lusaka in the last month? (circle one)	Y	N	If Yes, fill in details below

Places Travelled To Outside of Lusaka

Use one line per trip and be specific e.g. country, or if within Zambia then district or at least Province)

Total number of days spent outside Lusaka in the last 1 month:	

Malaria History (in last 1 month)

Has this person had any Malaria in the last month? (circle one)	Y	N	If Yes, fill in details below

Treatment Taken (tick all that apply)				Diagnosed by (tick all that apply)		
Coartem	Fansidar	Quinine	Other (specify)	RDT	Microscopy	Clinical

Patient Household Location and Contact Information

Physical Address (include landmarks as required)	
Contact Telephone Number	

Response to Index case

Net given to patient (circle one)	Y	Response required (circle one)	Y
	N		N
