# Supplementary material for: The use of spatial and genetic tools to assess Plasmodium falciparum transmission in Lusaka, Zambia between 2011 and 2015
Source: Malar J. 2020 Jan 15;19:20. doi: 10.1186/s12936-020-3101-7 (PMC6964105; doi:10.1186/s12936-020-3101-7)
Supplement: Supplementary file 3 — Additional file 3: Table S1. Characteristics of incident and RCD positive individuals with and without a travel history. [file 12936_2020_3101_MOESM3_ESM.docx]

# Additional Data

Additional file 3: Table S1: Characteristics of incident and RCD positive individuals with and without a travel history.

| **Variable** | **Travel (n=34)** | **No Travel (n=15)** |
| --- | --- | --- |
| Age (median [IQR]) | 17.98 [5.88 – 27.12] | 9.59 [2.99 – 22.93] |
| Sex (% male [95% CI]) | 52.94 [35.13 – 70.22] | 33.33 [11.82 – 61.62] |
| Incident case (% [95% CI]) | 97.06 [84.67 – 99.93] | 86.67 [59.54 – 98.34] |
| Polyclonal (% [ 95% CI]) | 38.24 [22.17 – 56.44] | 13.33 [1.66 – 40.46] |
| Relatedness (median [IQR]) | 73.91 [67.5 – 78.57] | 75.00 [70.00 – 82.35] |
| Symptoms (% [95% CI]) |  |  |
| Fever | 82.35 [65.47 – 93.24] | 73.33 [44.90 – 92.21] |
| Headache | 79.41 [62.10 – 91.30] | 53.33 [26.59 – 78.73] |
| Cough | 29.41 [15.10 – 47.48] | 20.00 [4.33 – 48.09] |
| Diarrhoea | 17.65 [6.76 – 34.53] | 26.67 [7.79 – 55.10] |
| Vomiting | 41.18 [24.65 – 59.30] | 46.67 [21.27 – 73.41] |
| Respiratory | 11.76 [3.30 – 27.45] | 26.67 [7.79 – 55.10] |
| Chest pain | 8.82 [1.86 – 23.68] | 13.33 [1.66 – 40.46] |
| Recent previous malaria (% [95% CI]) | 17.65 [6.76 – 34.53] | 0.00 [0.00 – 21.80] |
